# Lymph Node Metastasis in Gastrointestinal Carcinomas: A View from a Proteomics Perspective

**DOI:** 10.3390/curroncol31080333

**Published:** 2024-08-02

**Authors:** Vaishali Jain, Puja Sakhuja, Anil Kumar Agarwal, Ravi Sirdeshmukh, Fouzia Siraj, Poonam Gautam

**Affiliations:** 1 Indian Council of Medical Research, National Institute of Pathology, New Delhi 110029, India; 2 Faculty of Health Sciences, Manipal Academy of Higher Education (MAHE), Manipal 576104, India; 3 Govind Ballabh Pant Institute of Postgraduate Medical Education and Research (GIPMER), New Delhi 110002, India; 4 Institute of Bioinformatics, International Tech Park, Bangalore 560066, India

**Keywords:** gastrointestinal carcinomas, lymph node metastasis, proteomics, micrometastasis, sentinel lymph node

## Abstract

Lymph node metastasis (LNM) is one of the major prognostic factors in human gastrointestinal carcinomas (GICs). The lymph node-positive patients have poorer survival than node-negative patients. LNM is directly associated with the recurrence and poor survival of patients with GICs. The early detection of LNM in patients and designing effective therapies to suppress LNM may significantly impact the survival of these patients. The rapid progress made in proteomic technologies could be successfully applied to identify molecular targets for cancers at high-throughput levels. LC-MS/MS analysis enables the identification of proteins involved in LN metastasis, which can be utilized for diagnostic and therapeutic applications. This review summarizes the studies on LN metastasis in GICs using proteomic approaches to date.

## 1. Introduction

The human digestive system is made up of the gastrointestinal tract (GIT), including the accessory organs of digestion (tongue, salivary glands, pancreas, liver and gallbladder) [1]. GITs are categorized into three groups based on their location in the GIT, i.e., (a) the upper gastrointestinal tract (UGIT), which includes the esophagus, stomach and duodenum; (b) the middle gastrointestinal tract (MGIT), including the jejunum and ileum; and (c) the lower gastrointestinal tract (LGIT), including the colon, rectum and other locations [2]. Gastrointestinal carcinomas (GICs) account for ~24% of the global cancer incidence and ~36% of all cancer-related deaths. According to GLOBOCAN data estimates for 2022, there were 4.9 million new cases of GICs and 3.3 million deaths globally in 2022 [3]. Considering factors like age and incidence rate, it has been estimated that new cases and deaths from GICs will increase to 7.5 and 5.6 million, respectively, by 2040 [4].

**Figure 1:**
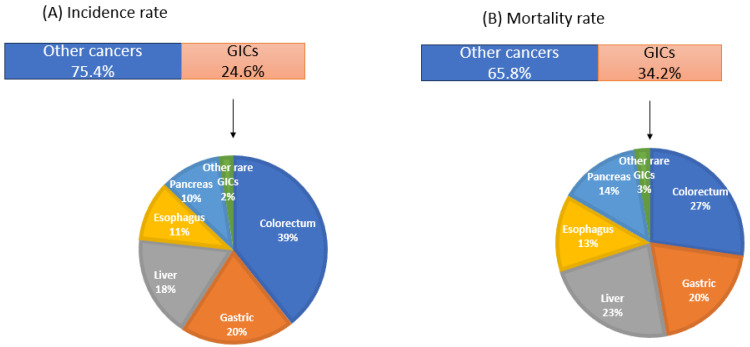
Global scenario of gastrointestinal cancers (GLOBOCAN data, 2022).

Among GICs, colorectal carcinoma is reported to have the highest incidence and mortality rates, followed by gastric, liver, esophagus, pancreas and gallbladder carcinoma [3] (Figure 1). Recurrence has been reported in these patients despite the standard treatment. Colorectal carcinoma is reported to have a recurrence rate of 16.6% in patients in stages I-III [5], gastric carcinoma with tumor recurrence in ~60% of early stages [6], hepatocellular carcinoma with overall ~26% recurrence [7], esophageal cancer with ~38% overall recurrence rate among the resectable cases [8], pancreatic cancer with ~52% early recurrence rate (i.e., recurrence within one year) [9] and gallbladder carcinoma with overall ~35% recurrence rate [10].

**Figure 2:**
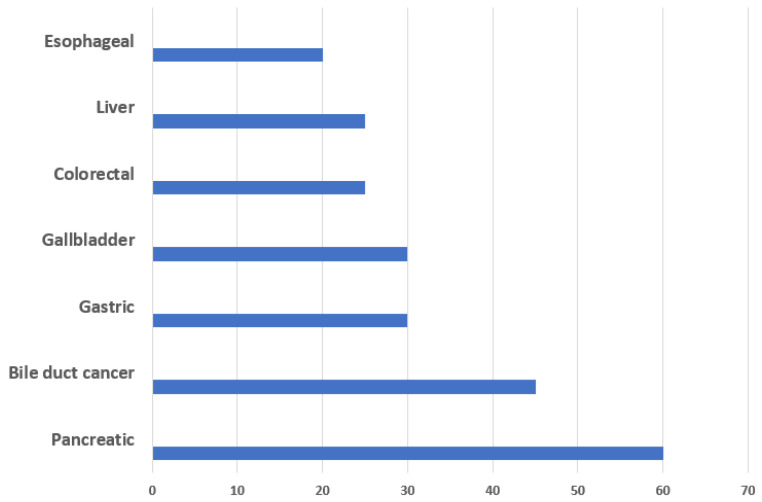
Frequency of lymph node metastasis in GICs.

Occult lymph node metastasis is one of the major factors associated with recurrence in GICs, leading to poor survival in the patients. Lymph nodes are the primary sites of spread for many solid tumors. The lymphatic system provides an easier route for tumor dissemination due to its incomplete basement membrane, single endothelial layer and low hydrostatic pressure [11]. Lymph node metastasis (LNM) is one of the major prognostic indicators in GICs [12]. The nodal staging in gastrointestinal cancers is shown in Table 1. The incidence rate of LNM varies in different cancers, with pancreatic and gallbladder carcinoma having the higher LNM rate, i.e., ~60% and ~30%, respectively (Figure 2). As patients with LN metastatic cancers are generally treated with chemotherapy in conjunction with adjuvants, differentiation between “localized” cancer (restricted to the primary tumor site) and “LN metastatic” cancer is useful for deciding the treatment regimen [13]. The size and number of metastatic LNs are the major risk factors of recurrence, and the evaluation of a number of LNs is reported to be a variable factor that affects prognostic outcome [14].

The sentinel lymph node (SLN), the initial station of lymphatic drainage from the primary tumor and the LN, is most likely to harbor metastasis. If the sentinel node is free of cancer cells, it is likely that the other nodes are also free of tumor cells. Dye-guided or radio-guided methods are being used for sentinel lymph node mapping. Blue-green dyes such as Evans blue, methylene blue, indocyanine green and radiocolloid are commonly used in the mapping of SLNs [15]. Indocyanine green dye was used intraoperatively before to map draining LNs and identify the SLN, but false-negative results have been reported in gastric cancer [16]. The dual tracer method combining both radiocolloid and blue dye is currently being used for mapping SLNs due to its higher detection rate and lower false-negative results; however, it is costly, invasive and employs the use of radioactive colloid [17,18]. SLNs are well-defined in breast cancer and melanoma; however, these are not clearly defined in GICs to date. Among GICs, sentinel nodes are relatively better defined in gastric cancer. A multicenter trial performed using both dye and RI (a dual tracer method) showed that the sensitivity to identify sentinel nodes was 98%, while the sensitivity to detect LN metastasis in gastric cancer was 93% [19]. Kitagawa et al. established a radio-guided intraoperative sentinel node navigation system using preoperative endoscopic submucosal injection of radioactive tracer followed by intra-operative gamma-probing. In 131 patients with GICs (esophagus: 22; stomach: 71; colorectum: 38), the detection rate of sentinel nodes was 91%, and the overall diagnostic accuracy of LNM by sentinel node status was 97% [20]. Overall, determining the SLNs in GICs could be useful for more precise staging in GICs and may significantly improve the survival outcome.

Micrometastasis (MM) is a cluster of 10 to 20 tumor cells or clumps of tumors measuring at least 0.2 mm in diameter [21]. Detection of nodal micrometastasis is extremely challenging for pathologists using only H&E staining. MM is the initial stage of cancer spreading through the lymphatic system, and if left undetected, it leads to the understaging of the cancer [21]. Generally, MM is detected by the presence of epithelial markers present in the lymph nodes, usually by cytokeratins (CKs), carcinoembryonic antigen (CEA), mucins and squamous-cell carcinoma-related antigen (SCC), but sometimes it is missed in the routine IHCs [22]. Examination of LN status is thus beneficial for accurate and precise staging, especially in pN0 patients; however, the major disadvantages include high cost and labor in processing lymph nodes through serial sectioning to search for malignancy [23]. Proteomics technology may be applied to identify novel biomarkers for the detection of MM in GICs, which might result in LN metastasis-specific markers useful for the detection of MM.

## 2. Lymphangiogenesis and the Role of Growth Factors and Chemokines

The process of lymphangiogenesis is mediated through the interaction between the cancer cell, tumor microenvironment and premetastatic site niche [24]. Tumor cells disseminate into lymphatic capillaries, and alterations in the functional properties of lymphatic endothelial cells (LECs) can promote adhesion and intravasation of tumor cells [25]. Vascular endothelial growth factors (VEGFs), specifically VEGF-C and VEGF-D, have been well studied in terms of their role in lymphangiogenesis. VEGF-C and VEGF-D are often expressed in primary human tumors and their associated stroma and are secreted by tumor cells, immune cells and tumor-associated fibroblasts [26]. Signaling between these VEGF-C/D and their receptors (VEGFR-2/3) present on lymphatic endothelial cells (LECs) drives the migration of tumor cells to lymph nodes [25]. Several studies revealed the correlation of higher VEGF expression with LNM in GICs and poor survival outcomes [27,28]. The emergence of monoclonal antibodies targeting VEGF has been proven to be effective in prolonging overall survival in patients with colorectal carcinoma patients [29]. Kabbinavar et al. observed improved response rates and overall survival in clinical trials when comparing patients treated with fluorouracil/leucovorin alone versus those treated with a combination of bevacizumab and fluorouracil/leucovorin [30]. Similarly, overexpression of VEGF-C/D increased lymphatic vessel quantity and density [28]. Other than VEGFs, the direct roles of chemokines and their receptors, CXCR3, CXCR4 and CCR7, have also been established in LN metastasis, contributing to the poor survival outcome [31,32]. Cancer cells overexpress these receptors and bind their respective ligands on lymphatic vessels within the primary tumor, resulting in the recruitment and patterning of several types of immune cells to lymph nodes [25]. Murakami et al. showed that the stable Knockdown studies reducing the expression of CXCR4 and/or CXCR4 significantly reduced LN metastasis in colorectal cancer in vivo. They further demonstrated that CXCR3 and CXCR4 work in a synergistic manner by studying in vivo metastatic effects after inoculating mice with double knockdown cells [31].

In addition to VEGFs and chemokines, several other proteins have also been reported to be associated with lymphangiogenesis in GICs. Ang-2 (angiopoietin 2) contributes to pancreatic ductal adenocarcinoma (PDAC) metastasis by promoting lymphatic vascularization and enhancing tumor cell interactions with endothelial cells [33]. The role of Eph/Ephrin is well studied in colon cancer, gastric and pancreatic cancer, and its overexpression is found to be correlated to lymphangiogenesis and tumor progression [34]. Fibroblast growth factors (FGFs) have been reported to induce lymphangiogenesis by targeting Akt/mTOR/p70S6K pathways in GICs [35]. Insulin-like growth factors are reported to be overexpressed in GICs and induce LNM by activating VEGFs [36]. Another protein, fatty acid-binding protein 5 (FABP5), has been reported to activate immune-related pathways, including cytokine–cytokine receptor interaction, interleukin-17 signaling, and tumor necrosis factor signaling, thereby stimulating LNM in gastric cancer [37]. Tumor necrosis factor-α enhances lymphangiogenesis in gallbladder carcinoma via nuclear factor-κB-mediated upregulation of VEGF-C [38].

Anti-VEGF therapies are reported to work well in some preclinical and clinical cases; however, these therapies are not effective for all tumors [39]. Tumors can develop specific mechanisms to evade anti-angiogenic therapy, which include upregulation of compensatory pathways, vasculogenic mimicry and recruitment of bone-marrow-derived cells [40]. Therefore, there is a need to develop new drugs that can be used alone or in combination with existing angiogenic therapies or chemotherapeutic therapies to manage the lymphatic progression of the cancer cells. Proteins are the ultimate effector molecules, and with the advancements in proteomics technologies, the detection of LN metastatic signatures in the primary tumors and lymph nodes may be possible, opening new paths toward molecular diagnosis and designing effective treatment strategies of cancers [41]. This article compiles the progress that has been made to understand LN metastasis in GICs to date, including esophageal, gastric, colorectal, hepatocellular, pancreatic and gallbladder carcinoma using quantitative proteomics analysis.

## 3. Proteome Profiling in GICs

Proteomics is an important high-throughput technology that may provide valuable information on the identification, expression levels and post-translational modification of various proteins. The development of proteomics technology has significantly improved the understanding of the biology of various diseases, including cancer. This technology is being used to understand the underlying molecular mechanism associated with lymph node metastasis in GICs and is reviewed here.

### 3.1. Colorectal Carcinoma

Colorectal carcinoma (CRC) is the third most common cancer worldwide in terms of incidence and reported to have more than 1.85 million cases and 0.85 million deaths annually [3]. Colorectal cancer is a multifactorial disease. Individuals who have colon polyps or inflammatory bowel diseases are at a higher risk for developing CRC [42]. Diagnostic modalities include a digital rectal exam (DRE), fecal immunochemical test for hemoglobin (FIT), guaiac fecal occult blood test (gFOBT), flexible sigmoidoscopy (FS), colonoscopy (OC), abdominal ultrasound, magnetic resonance imaging and computed tomographic (CT) scans. Carcinoembryonic antigen (CEA) is reported to be elevated in patients with CRC [42]. The treatment for LN-negative cases includes surgical resection, while in LN-positive cases, surgery and adjuvant therapy are suggested [43]. The recurrence has been detected in around 20–30% of the LN-negative cases with complete resection, suggesting that this might be due to the occult LN metastasis [44]. Left-sided T1 CRC, i.e., sigmoid colon and rectum, exhibited higher rates of LNM than right-sided T1 CRC, followed by higher rates of lymphatic invasion [45]. Left-sided location is an independent risk factor for LNM. Overall, the 5-year survival rate is reported to be 30–60% in patients with LN metastasis in contrast to 70–80% with LN-negative disease [44].

### 3.2. Proteomics in LN Metastatic Colorectal Carcinoma

Several high-throughput proteomic studies have been carried out to understand LNM in colorectal cancer, which are shown in Table 2. The majority of the studies were undertaken using primary tumor tissues, while one of the studies was carried out using LN tissue, and one study analyzed serum samples. In one study, two-dimensional gel electrophoresis (2-DE) coupled with MALDI-TOF-MS revealed the higher expression levels of HSP-27, Annexin II and Glutathione S transferase (GST) in the primary tumor of LN metastatic CRC patients [46]. In another study, 2D-DIGE followed by MS analysis led to the identification of elevated levels of transgelin in LN-positive CRC patients. Further, the miRNA-mediated knockdown of transgelin in human colon carcinoma cell lines HCT116 and SW480 led to a decrease in invasion and metastatic characteristics of cells and reduced the clonogenic survival and the percentage of viable cells [47]. Ma et al. carried out a 2D analysis followed by mass spectrometric analysis and reported the association of Cathepsin D, ubiquitin C-terminal hydrolase 1 (UCH-L1) and ferritin heavy chain (FHC) with LNM [48]. Another study by Mori et al. demonstrated the higher expression levels of ezrin in LN-positive CRC using iTRAQ-based proteomic approaches and subsequently validated its expression at protein as well as mRNA levels in an independent cohort of samples using IHCs and RT-PCR, respectively. Attenuation of the expression of ezrin inhibited the invasion and migration of cells in vitro [49]. In a recent study, MS-based analysis identified higher levels of gelsolin and peroxiredoxin 4 in LN-positive CRC and established their role in metastasis in vitro and in vivo [50]. Lee et al. identified upregulated expression levels of ubiquitin carboxyl-terminal hydrolase isozyme L1 (UCHL1) and chromogranin-A (CHGA) in the primary tumor tissue of LN metastatic CRC patients using iTRAQ-based proteomic analysis and explored its role in invasion, migration and reactive oxygen species generation in cell lines [51]. He et al. analyzed the proteome of sentinel lymph nodes by 2DE coupled with MaLDI-TOF-MS and revealed the higher expression of Annexin A1, hnRNP A1, ezrin and Tubulin b-2C in sentinel lymph nodes of LN-positive CRC patient [52]. We have carried out gene ontology and pathway analysis of DEPs found in primary tumor vs. lymph node tissue in colorectal cancer using Reactome pathways (Supplementary Figure S1). Gene ontology studies reveal ‘cell adhesion proteins’ among the top molecular functions, while ‘lipid binding proteins’ were among the top molecular functions in lymph nodes. ‘Regulation of IGF transport and uptake by IGFBP’ was among the top altered pathways in LN (including APOA1, TF, SERPINA1), while ‘signal-recognition particle (SRP)-dependent co-translational protein targeting to membrane’ (including majorly ribosomal proteins) was among the top altered pathway in the primary tumor. None of the studies included matched primary tumor and lymph node tissue, which may provide a better understanding of the LN metastasis in colorectal cancer.

For the tissue-based proteomic studies, the validations were undertaken in a larger cohort of clinical samples (primary tumor and/or metastatic LNs). The proteins Annexin A1, hnRNP A1, ezrin and Tubulin β-2C were identified in the sentinel lymph nodes (LNs) of colorectal cancer (CRC) patients. Subsequently, their presence was validated in a larger cohort of LNs, providing strong evidence for their potential utility in future applications [52]. Similarly, the proteins Cathepsin-D, UCHL-1, and FHC were validated in a small set of lymph nodes [50]. Their functional characterization in a cell line could draw more attention to these proteins. GSN and PRDX4 were initially validated in a smaller cohort. However, their subsequent functional validation in both in vitro and in vivo settings positions them as promising markers for further exploration of these proteins in clinical settings. The suppression of tumorigenesis in a mouse model suggests that UCH-L1 could be a promising marker for future studies [51]. Further investigation into its role and potential applications may yield valuable insights for cancer research and treatment. The analysis of TTR (transthyretin) in serum samples enhances its potential as a circulatory biomarker for the detection of LN metastasis [53].

The establishment of a pair of colorectal cell lines SW480 (Dukes B carcinoma) and its lymph node derivative SW620 (Dukes C carcinoma) from the same patient offered a great opportunity to study proteins associated with LNM [54]. These cell lines were exploited in different ways to study LNM. Comparative proteomics analysis of SW480 and SW620 revealed 11 DEPs, of which HSP27 was validated in primary colorectal cancer patients using IHCs [55]. Xue et al. analyzed the secretome of these cell lines and showed the upregulation of two proteins, Trefoil factor 3 (TFF3) and Growth/Differentiation Factor 15 (GDF15), in serum and tissues of CRC patients [56]. iTRAQ-based quantitative proteomics of cell lysates of SW480 and SW620 revealed the overexpression of calcylin-binding protein (CacyBP) and downregulation of β-catenin in metastatic cell lines [57]. Proteomic profiling of extracellular vesicles derived from SW480 and SW620 has been carried out, and cell adhesion-related proteins were found to be enriched in SW480, while cancer progression proteins were enriched in SW620 [58].

**Table 2:** Proteomic studies analyzing LN metastasis in colorectal carcinoma.

**(A) Tissue/serum-based studies**						
**Reference**	**Proteomics Approach/Samples**	**DEPs**	**Validation Method/Samples**	**Validated Proteins**	**Result**	**Functional Characterization**
[46]	2DE LC MS/MS FFr tissue (primary tumor/ 5 LN positive, 5 LN negative)	25 (Mascot score > 63, *p* < 0.05)	Western blot and IHC/ FFPE primary tumor 40 LN negative and 40 LN positive	HSP-27, GST, Annexin II, L-FABP	HSP-27, GST and Annexin II upregulated in LN-positive patients L-FABP downregulated in LN-negative patients	NA
[47]	2D DIGE, MS/FFr tissue (primary tumor) 12 LN negative and 12 LN positive	6 (FC > 2)	IHCs using TMAs/FFPE primary tumor 48 LN negative and 46 LN positive	Transgelin	Upregulated in LNM	miRNA-mediated knockdown in two cell lines. Decreased invasion and metastatic characteristics of cells and reduced the clonogenic survival and the percentage of viable cells.
[59]	2 DE, MALDI-TOF MS/MS/FFr tissue (primary tumor) 6 LN negative and 6 LN positive	12 (FC > 1.5)	Western blot and IHCs/FFPE primary tumor 46 LN negative 37 LN positive	TCPZ and PPIB	TCPZ was downregulated PPIB was upregulated	siRNA mediated knockdown of PP1B in SW480 cells. Inhibited cell migration, invasion and the inhibition of closure rate
[52]	2DE, MALDI-TOF-MS/FFr tissue (lymph node) 62 normal LN and 126 sentinel lymph nodes from 43 patients	40 (FC > 2)	Western blot, IHC/FFPE lymph nodes 62 normal LN and 126 sentinel lymph nodes from 43 patients	Annexin A1, hnRNP A1, ezrin, Tubulin b-2C	All 4 proteins upregulated in sentinel lymph nodes	NA
[48]	2DE, MALDI-TOF-MS/FFr tissue (primary tumor) FFr, 5 LN positive and 5 LN negative	33 (FC ≥ 2)	Western blots and IHCs/FFPE 27 normal colorectal mucosa, 65 primary CRC and 26 positive LNs	Cathepsin D, UCH-L1 and ferritin heavy chain (FHC)	Cathepsin D, UCH-L1, upregulated in LNM FHC downregulated in LNM	In vitro Overexpression of UCH-L1, resulting in increased invasiveness of HCT8 cells.
[60]	LFQ, MS/FFr tissue (primary tumor) 3 LN negative and 3 LN positive	28 (FC > 2, *p* < 0.05)	IHCs/FFPE 168 primary colon cancer (87 LN negative and 81 LN positive)	FXYD3, S100A11, GSTM3	FXYD3, S100A11, GSTM3 Upregulated in LNM	NA
[61]	LFQ LC-MS/MS/FFr tissue (primary tumor) 9 LN negative and 10 LN positive	29 Using R package Local FDR < 0.15	IHCs/FFPE primary 20 LN negative and 20 LN positive	MX1, IGF1-R and IRF2BP1	MX1 and IGF1-R upregulated in LNM; IRF2BP1 downregulated in LNM	In vitro siRNA mediated knockdown of MX1. MX1 knockdown strongly inhibits wound healing of DLD1 cells.
[62]	iTRAQ LC-MS/MS/FFr tissue (primary tumor) 5 LN positive, 5 LN negative	60 (FC < 0.5)	Western blots and IHCs/FFPE primary 54 LN positive 103 LN negative	HSP47	HSP47 upregulated in LNM	NA
[49]	iTRAQ LC-MS/MS/FFr tissue (primary tumor) 5 LN positive, 5 LN negative	55 (FC < 0.75)	IHCs and RTPCR Cohort 1 IHC FFPE 82 LN positive and 113 LN negative Cohort 2 RTPCR FFr primary (63 LN positive and 107 LN negative)	Ezrin	Ezrin upregulated in LNM	In vitro siRNA mediated ezrin knockdown in DLD1 and LoVo cells. Ezrin contributes to the migration and invasion capacity of CRC cells.
[50]	2D-DIGE MS/ FFr tissue (primary tumor) 8 LN negative 8 LN positive	18 (FC > 1.5)	IHCs/FFPE primary (18 LN negative and 22 LN positive)	Gelsolin, peroxiredoxin 4	Both proteins are overexpressed in LNM	In vitro and in vivo Silencing of GSN and PRDX4 by lentiviral shRNA induces cell cycle arrest and decreases migration and invasion of DLD-1 cells.
[51]	iTRAQ 2D LC-MS/MS FFr tissue (primary tumor) 12 LN negative 12 LN positive	48 (FC > 1.5)	IHCs/FFPE primary 60 LN negative 56 LN positive	Ubiquitin carboxyl-terminal hydrolase isozyme L1 (UCH-L1) chromogranin A e (CHGA)	Upregulated in LNM	In vitro and in vivo Silencing of UCH-L1 and CHGA induced cell cycle arrest and decreased migration and invasion. Suppressed tumorigenesis in vivo.
[53]	2D, MS/serum 32 LN negative; 40 LN positive	8 (FC > 2, *p* < 0.05)	ELISA/86 serum samples	Transthyretin (TTR)	Downregulated in LNM	NA
**(B). Cell line-based studies**						
**Reference**	**Proteomics Approach/Samples**	**DEPs**	**Validation Method/Samples**	**Validated Proteins**	**Result**	**Functional Characterization**
[55]	2D MALDI TOF/Cell lines SW 480 AND SW620	11 (FC > 2, *p* < 0.05)	Western blot, RT PCR and IHCs/FFPE primary: 30 LN positive and 38 LN negative	HSP27	HSP27 overexpression in LNM patients	NA
[56]	LC-MS/MS/Secretome of SW 480 and SW620	145 (FC > 1.5)	ELISA and IHCs/ serum of 76 LN positive, 68 LN negative and 156 healthy For IHCs, 31 FFPE LN negative 38 LN positive	ELISA: TFF3 AND GDF15 IHC: TFF3 AND GDF15 For ELISA	Elevated expression of GDF15 and TFF3 in LN-positive CRC patients	NA
[57]	iTRAQ-based LC-MS/MS/ cell lysate SW480 and SW620	147 (FC 1.5, *p* < 0.05)	Western blot RT-PCR/ cell lines	CacyBP and β-Catenin	CacyBP upregulated in SW620 β-Catenin downregulated in SW480	Overexpression of CacyBP in primary colon cancer cell lines showed downregulated levels of cellular β-Catenin and significant reduction in cellular adhesion.
[58]	LFQ LC-MS/MS/ EVof SW480 and SW620			Only profiling has been carried out SW480 EV-enriched proteins: 368 SW620 EV-enriched proteins: 359 (FC > 1.5). No validations	Gene ontology studies undertaken	NA

### 3.3. Gastric Carcinoma

Gastric carcinoma (GC) is the sixth most common cancer in the world and the fourth leading cause of death worldwide [3]. It is reported to be more frequent in men. *Helicobacter pylori* and Epstein–Barr virus (EBV) infections and chronic atrophic gastritis are the major risk factors for GC. Upper gastrointestinal endoscopy and biopsy remain the gold standard for the diagnosis of GC. Other diagnostic methods include imaging strategies such as computed tomography (CT), magnetic resonance imaging (MRI) and positron emission tomography (PET) [63]. Elevated serum levels of CA 19-9, CEA, carbohydrate antigen 72-4 (CA 72-4) and carbohydrate antigen 15-3 (CA 15-3) are the most commonly used biomarkers for GC [64]. Surgical intervention is an essential component of the therapeutic approach for GC. Preoperative chemotherapy using 5-fluorouracil (5FU) or cisplatin and radiation therapy is useful for treating advanced-stage GC. Targeted therapy against VEGFR2 and HER2 (ramucirumab and trastuzumab, respectively) is also being used after the first line of treatment fails [63]. The 5-year survival rate of patients with LN metastasis is reported to be <30% [65].

### 3.4. Proteomics in LN Metastatic Gastric Carcinoma

There are few high-throughput proteomics studies available that focus on understanding LNM in gastric cancer, as listed in Table 3 and discussed below. Jung et al. identified 12-fold higher levels of galectin-2 in LN-negative primary tumors of GC patients and subsequentially validated them in a larger cohort of samples [66]. Galectin-2 is primarily expressed by gastrointestinal epithelial cells. Notably, it is found in mucous neck cells and surface mucous cells of the stomach, as well as in goblet cells within the small intestine [67]. However, its validation in lymph nodes and functional characterization needs to be undertaken for more clinical value.

Serum analysis from gastric cancer patients with LNM and without LNM revealed that 85.4% of those with LNM had a positive expression for Fibrinopeptide A with alanine truncation at the N-terminal (degAla-FPA, 1465.63 Da), as determined by tandem mass spectrometry in combination with magnetic beads [68]. Their findings supported the fact that FPA exists in gastric cancer patients in a hypercoagulative state and could be useful as a prognostic biomarker. Additionally, 2-DE coupled with MALDI–TOF/TOF–MS analysis revealed profilin-1 to be downregulated and 14-3-3β to be upregulated in node-positive GC tissue [69].

### 3.5. Hepatocellular Carcinoma

Liver cancer is the seventh most common cancer in the world and the third most common cause of death [3]. Hepatocellular carcinoma (HCC) accounts for around 80% of liver cancers, and the major predisposing risk factors for HCC include viruses [Hepatitis B virus (HBV) and hepatitis C virus (HCV)], alcoholic cirrhosis and non-alcoholic fatty liver disease (NAFLD) [70]. Complete blood tests, elevated levels of alkaline phosphatase (ALP), alpha-fetoprotein (AFP) and PIVKA-II (Protein-Induced Vitamin K Absence or Antagonist-II) are the major diagnostic methods for HCC [71]. Surgical resection is recommended for localized HCC; however, the recurrence rate after five years is very high. Liver transplantation is considered in case of liver dysfunction, portal hypertension, or multi-tumor involvement, while the systemic treatment with lenvatinib, regorafenib, cabozantinib, ramucirumab and the anti-PD-1 antibodies (nivolumab and pembrolizumab) are available for advanced HCC [72]. The median survival time was found to be 28 months in the patients with LN metastasis and 53 months in the patients without LN metastasis. The recurrence rate was reported to be 82% in LN-positive patients than 57% in node-negative disease [73]. No high-throughput proteomic studies have been carried out to date to understand LN metastasis in HCC. Therefore, it needs to be explored for better prognosis and treatment strategies.

### 3.6. Esophageal Carcinoma

Esophageal cancers rank tenth in terms of incidence rates worldwide and contribute to 5.5% of all global cancer deaths [3]. Major risk factors include alcohol and smoking [1]. EUS, CT scan and MRI are the imaging modalities commonly being used to screen esophageal carcinoma [74]. Surgical resection and chemoradiotherapy are commonly used for treatment. Combined therapy with cisplatin+5-FU is currently being used as the first line of treatment for patients with advanced esophageal carcinoma [75]. The targeted therapy, including cetuximab and bevacizumab (EGFR and VEGF, respectively), trastuzumab (monoclonal antibody against HER-2) and pembrolizumab (PD-L1), is being used in the management of the advanced stage of disease [76]. The 5-year survival rates were reported to be 59.8%, 33.4% and 9.4% in the patients with 0, 1 and ≥2 metastatic LN, respectively [77]. The mechanism underlying LNM is poorly understood, and no high-throughput proteomic study has been performed to date.

### 3.7. Pancreatic Carcinoma

Pancreatic cancer is the fourteenth-ranked cancer in terms of incidence and is the seventh leading cause of cancer deaths worldwide, suggesting that this cancer remains a threat to public health [3]. Men are at a higher risk of developing pancreatic cancer than females due to lifestyle habits such as smoking and tobacco use [78]. The current screening methods include CT scan, magnetic resonance cholangiopancreatography (MRCP) and endoscopic ultrasound (EUS) [79]. Surgery is the gold standard treatment for pancreatic cancer, and for advanced disease, chemotherapeutic combinations including FOLFIRINOX (5-fluorouracil, folinic acid [leucovorin], irinotecan and oxaliplatin) and gemcitabine plus nab-paclitaxel are available [80]. The median overall survival rates were reported to be 25.5 months, 21 months and 12.3 months in the patients with 0, 1–2 and >3 metastatic LNs [81].

### 3.8. Protoeomics in LN Metastatic Pancreatic Carcinoma

The studies related to LN metastasis in pancreatic cancer are shown in Table 4. In a study by Cui et al., radixin, moesin and c14orf166 were found to be significantly upregulated in LNM pancreatic tumor tissues using 2D-DIGE followed by MS/MS analysis and validation by IHC analysis [82]. Naidoo et al. carried out mass spectrometric analysis on primary tumors and LNs of pancreatic cancer and revealed the overexpression of S100P and stratifin in LNs as compared to primary tumors [83]. These two proteins were further validated by IHC analysis. Suzuki et al. carried out label-free quantitative proteomics and identified the higher levels of hemopexin in LN-positive pancreatic cancer tissue and established its role in migration and invasion in vitro [84]. Overall, the proteomic studies revealed the association of radixin, moesin, c14orf166, S100P and hemopexin in LN metastasis in pancreatic cancer.

### 3.9. Other Rare GICs

The other rare GICs include gallbladder carcinoma, bile duct cancer, small intestine and anal cancer. GBC is a rare cancer and is the sixth most frequent malignancy of the GI tract [85]. Gallstone disease (GSD) is a major risk factor for GBC and has been reported in 70–94% of patients with GBC [85]. Other risk factors included the presence of chronic infection of the gallbladder with *Salmonella typhi* or *Helicobacter* sp. [86]. GBC is more common in women. The five-year survival rate of GBC cases with node-negative disease was approximately 80%, while for node-positive pN1 and pN2 was around 57% and 23%, respectively [87,88]. In a study conducted by Amini et al., none of the patients who had more than four positive LNs could survive beyond 5 years [89]. Agarwal et al. reported that a routine 16b1 LN biopsy (interaortocaval lymph node) and intraoperative frozen section evaluation might avoid radical surgery in ~20% of resectable GBC patients [90]. ‘Bile duct cancer’, also known as cholangiocarcinoma, is a rare malignancy that originates in the slender tubes called bile ducts. Primary sclerosing cholangitis, chronic liver disease, bile duct abnormalities and liver fluke infection are the major risk factors associated with cholangiocarcinoma [91]. Patients with nodal disease have a less than 5-year survival rate (~9%) compared with those having no nodal disease (26%) [92]. Small intestine cancer is a rare malignancy originating from the small bowel. The 5-year disease-free survival rate has been reported to be lower in node-positive disease (~30%) than in node-negative disease (~48%) [93]. Anal cancer is an uncommon type of cancer, generally transmitted through sexual contact. Human papillomavirus (HPV) is a common cause [94]. The 5-year survival rate of anal cancer with LNM is worse than that of patients with LNM (66% vs. 82%) [95].

### 3.10. Proteomics in LN Metastatic Rare GICs

Our group has carried out iTRAQ-based quantitative proteomics and identified 58 differentially expressed proteins (DEPs) specifically in LN-positive GBC. They reported a significant overexpression of Keratin 7 (KRT7) and sorcin (SRI) in LN metastatic GBC cases using Western blot and IHC analysis [96] (Table 5). Further, the knockdown of SRI significantly inhibited the cell proliferation, invasion and migration and regulated the epithelial-to-mesenchymal transition (EMT) of GBC cells, suggesting sorcin as a novel regulator and a therapeutic target for patients with LN-positive GBC (unpublished data). LNM is an essential prognostic factor for patients with small intestine cancer (cholangiocarcinoma) [92,93]; however, the mechanisms underlying LNM have not been explored in these cancers. No high-throughput proteomics studies have been done in these cancers.

## 4. Discussion and Future Perspectives

Lymph node metastasis is one of the major prognostic factors for the GICs and a major contributor to mortality in these patients. Proteins play a crucial role in the LNM of different cancers. Mass spectrometry is a cutting-edge technology that enables researchers to identify biomolecules that can be used as diagnostic or prognostic biomarkers or as drug targets for therapeutic applications. There have been significant advances in MS techniques, and proteomic approaches have been used to explore the molecular mechanism associated with LNM in GICs. Here, we have compiled the data from high-throughput proteomic studies carried out to understand LNM in GICs.

Initially, we collected the literature, including high-throughput proteomic studies, to understand the molecular processes associated with LNM in GICs. A non-redundant list of differentially expressed proteins in LN metastatic cancer was prepared for each GIC (Supplementary Table S1). In order to understand the molecular functions and pathways associated with LNM-specific proteins in GICs, STRING analysis was performed using the LNM-specific proteins identified from various proteomic studies for each GIC. ‘Structural constituent of Cytoskeleton”, “Cell adhesion molecule binding”, ‘cytoskeleton-binding’ or ‘cadherin-binding’ proteins were among the top-ranked molecular functions (Supplementary Figure S2). “Regulation of actin cytoskeleton” was among the top-ranked molecular pathways in gastric and pancreatic cancer, ‘propanoate metabolism’ in colorectal cancer and ‘fatty acid degradation’ in gallbladder carcinoma (Supplementary Figure S3). Some of the common cytoskeleton proteins involved in the LNM of GICs were transgelins and annexins (Table 2, Table 3, Table 4 and Table 5). Transgelin is a 22 kDA actin-binding protein belonging to the calponin family. It is involved in smooth muscle differentiation and is also associated with Ca^2+^-independent smooth muscle contraction [97]. Transgelin has been reported to increase the migratory ability and invasive potential of tumorigenic cells and enhance EMT via activating STAT3 signaling [98,99]. The Annexins belong to the family of calcium-regulated phospholipid-binding proteins and are reported to promote the invasion and migration of cancer cells and the progression of the disease [100]. Another common protein associated with LNM in GICs was heat shock proteins. HSP27, a small HSP that is regulated both transcriptionally and post-translationally, plays a role in modulating the polymerization and reorganization of actin filament and mediates EMT in various cancers [101].

Some of the proteins identified from the proteomic studies showed a significant correlation with poor prognosis. The overexpression of HSP47 in LN-positive patients with CRC is associated with tumor progression and poor prognosis [62]. The expression levels of 14-3-3β and profilin-1 proteins were associated with various factors, including lymph node metastasis, and they also predicted the overall survival of patients with gastric cancer [69]. Various other studies have indicated the overexpression of ANXA1, cathepsin D, FXYD3, S100A11, PRDX4, GDF15 and TFF3 is associated with poor overall survival and prognosis in CRC patients [102,103,104,105,106,107,108]. Moesin and stratifin are associated with poor overall survival in patients with pancreatic cancer [109,110]. These proteins could serve as a promising biomarker for prognosis in GICs, potentially opening new therapeutic avenues.

LN metastasis in patients results in poor prognosis as recurrence is common in these patients. As mentioned earlier, one of the major causes of recurrence is occult LN micrometastasis (MM), and detection of MM can help to determine the stage of cancer more precisely. Unfortunately, commonly used pathological examination may miss the detection of micrometastasis in lymph nodes due to the presence of a low number of tumor cells. It is thought that the molecular biomarkers predicting LNM might lead to a better detection rate than conventional histopathological evaluation, and clinicians might use these biomarkers to identify LNM. At present, epithelial markers, such as CK7 (cytoplasmic proteins), CEA and mucins (membrane proteins), are reported to detect micrometastasis in GICs; however, it is technically challenging to detect a few tumor cells present in the lymph nodes. Detection of LNM using non-invasive blood-based markers may be highly useful in improving the survival outcome; however, there are limited serum/plasma studies with respect to this field, and more proteomics-based studies are needed to achieve a panel of markers for this purpose.

Angiogenesis and lymphangiogenesis have common mechanisms and pathways, including the VEGF/VEGF-R axis. In order to restrict the tumor spread to lymph nodes, only the VEGF/VEGF-R axis has been exploited, and antibodies against VEGF/VEGF-R and tyrosine kinase inhibitors are in use [111]; however, resistance against these drugs are reported in various cancers [112,113]. Therefore, it is important to design lymphangiogenesis-specific therapeutic targets to restrict metastasis to lymph nodes, improving patients’ overall survival. Furthermore, it is worth considering the exploration of natural inhibitors targeting these proteins. Additionally, the design of drug inhibitors could facilitate testing the efficacy of these molecules in clinical settings. Peptide aptamers can be designed to specifically bind to target molecules with high affinity. They can be used to modulate the function of target proteins and can inhibit protein–protein interactions affecting cellular processes.

Proteomics plays a crucial role in understanding complex biological processes. When it comes to studying LNM, proteomic techniques face several challenges. Lymph nodes are intricate structures with diverse cell types, including immune cells, stromal cells and cancer cells. Analyzing proteins from such heterogeneous samples is challenging. Laser Capture Microdissection (LCM) is a powerful technique used to selectively isolate specific cell populations from heterogeneous tissues and offers spatial resolution. It is useful in studying protein expression patterns within specific tissue regions. However, this technique requires specialized equipment. Due to the small amount of captured material, protein yield can be limited [114]. Single-cell proteomics is an exciting and rapidly advancing field that aims to understand the diversity of protein expression within individual cells. Unlike traditional proteomics, which analyzes bulk samples containing thousands of cells, single-cell proteomics focuses on characterizing proteins at the level of individual cells. Single cells contain minimal material, demanding ultra-sensitive techniques. Digital Spatial Profiling (DSP) is a cutting-edge technology that allows researchers to profile tissues for both mRNA and protein expression with spatial context. It can interrogate a large number of biomarkers simultaneously. Like many emerging technologies, DSP can be expensive, limiting its accessibility to some laboratories. Data generated from proteomic experiments are vast and complex. Analyzing these data requires robust computational tools. Researchers need to handle missing data, normalize across samples and account for batch effects. The lack of common standards for data representation and analysis hinders comparison across studies. Integrating proteomics with genomics, transcriptomics and clinical data enhances insights. Validating proteomic findings using orthogonal methods (e.g., Western blot, immunohistochemistry) is resource-intensive. Therefore, identifying reliable biomarkers for LNM prediction remains a challenge.

## 5. Conclusions

Overall, proteomic studies are majorly carried out in colorectal cancer, pancreatic cancer and gastric and gallbladder cancer, while similar studies are not yet performed in other GICs such as esophageal and hepatocellular carcinoma. These studies have provided a better understanding of the proteins and molecular mechanisms associated with LNM in different GICs. The majority of these studies used either the primary tumor specimens or the lymph node specimens; however, future studies may consider analyzing matched primary tumor and metastatic LNs, which will provide a better insight into the processes associated with LN metastasis, enabling personalized and more effective treatment strategies. The clinical verification of the promising proteins identified in the proteomic studies needs to be further validated in a large cohort of clinical samples, which might result in the tissue/blood-based test for early detection of LNM cases. Further, the functional validation of these proteins in vitro and in vivo may be explored for therapeutic applications in LN metastatic GICs. Future studies analyzing both tumor and immune cells in primary LNM using single-cell proteomics may be useful for gaining better insight into the molecular processes associated with LNM. In the long run, these insights will assist in identifying new molecular targets that could be used to impede LN metastasis and improve patient survival in GICs.

## Figures and Tables

**Table 1:** Nodal staging in GICs.

S. No.	Cancer	Description
1.	Colorectal Carcinoma	N1a—metastasis in 1 regional LN N1b—metastasis in 2–3 regional LNs N1c—metastasis in the regional lymph nodes does not contain cancer, but the cancer cells are in the tissue near the tumor N2a—metastasis in 4–6 regional LNs N2b—metastasis in ≥ 7 regional LNs
2.	Gastric Carcinoma	N1—metastasis in 1–2 regional LNs N2—metastasis in 3–6 regional LNs N3a—metastasis in 7–15 regional LNs N3b—metastasis in ≥15 regional LNs
3.	Hepatocellular Carcinoma	N1—metastasis in ≥1 regional LN
4.	Esophageal Carcinoma	N1—metastasis in 1–2 regional LNs N2—metastasis in 3–6 regional LNs N3—≥7 regional LNs
5.	Pancreatic Carcinoma	N1—metastasis in 1–3 regional LNs N2—metastasis in ≥4 regional LNs
6.	Gallbladder Carcinoma	N1—metastasis in 1–3 regional LNs N2—metastasis in ≥4 regional LNs
7.	Bile Duct Cancer	N1—metastasis in 1–3 regional LNs N2—metastasis in ≥4 regional LNs
8.	Small Intestine Cancer	N1—metastasis in 1–2 regional LNs N2—metastasis in ≥3 regional LNs
9.	Anal Cancer	N1—metastasis in nearby lymph nodes

**Table 3:** Proteomic studies analyzing LN metastasis in gastric carcinoma.

Reference	Proteomics Approach/Samples	DEPs	Validation Method/Samples	Validated Proteins	Result	Functional Characterization
[66]	ICAT labeling LC-MS/MS/ FFr tissue (primary tumor) 4 LN positive 3 LN negative	151 (FC > 1.5, *p* < 0.05)	Western blot and IHC/120 FFPE primary tumor	Galectin -2	Downregulated expression of Galectin-2 in LNM	NA
[68]	2DE, LFQ MS/MS/ FFr tissue (primary tumor) 12 LN positive 12 LN negative	26 (Mascot score > 60)	Validations by IHC/FFPE 43 LN negative and 85 LN positive	14-3-3β and profilin-1	14-3-3β protein upregulated in LNM Profilin-1 downregulated in LNM	NA
[69]	LC-MS/MS/ Serum 33 LN-negative patients and 157 LN-positive patients	NA	Not undertaken	Fibrinopeptide A (FPA) with alanine truncation at N terminal	85.4% of patients with LNM had FPA with alanine truncation at the N-terminal	NA

**Table 4:** Proteomic studies analyzing LN metastasis in pancreatic carcinoma.

Reference	Proteomics Approach/Samples	DEPs	Validation Method/Samples	Validated Proteins	Result	Functional Characterization
[82]	DIGE, MS/FFr tissue (primary tumor) 8 LN positive and 7 LN negative	33 (FC > 2, *p* < 0.05)	Western blot and IHC/63 pancreatic cancer tissues (37 LN positive and 26 LN negative) and 11 normal pancreas tissues	Radixin, moesin, ezrin and c14orf166	Radixin, moesin and c14orf166 upregulated in LNM Ezrin—no significant results	NA
[83]	LFQ LC-MS/MS/ FFPE tissue (primary tumor) 7 LN negative 7 LN positive	115 g test (g-value ≥ 3.8)	IHCs/55 FFPE tissues of matched primary PDAC and LN metastases (LNs)	S100P, moesin, stratifin	S100P and stratifin significantly upregulated in LNM Moesin—no significant results	NA
[84]	LFQ LC-MS/MS/FFPE tissue (primary tumor) 5 LN positive and 5 LN negative	9 (log2 ratio, >1 or <0.05)	IHCs/FFPE tissue (primary tumor) 5 LN positive and 5 LN negative	Hemopexin FTL	Hemopexin expression was associated with LN metastasis, whereas FTL expression was not associated	In vitro Stimulant hemopexin given It promotes invasion and migration of pancreatic cancer cells

**Table 5:** Proteomics studies analyzing LN metastasis in gallbladder carcinoma.

Reference	Proteomics Approach/Samples	DEPs	Validation Method/Samples	Validated Proteins	Results	Functional Characterization
[96]	iTRAQ labeling LC-MS/MS/ FFr tissue (primary tumor) 4 gallstone disease cases, 3 LN negative and 4 LN positive	58 (FC ≥ 2, *p* < 0.05)	Western blotting and IHCs/FFPE primary tumor of 15 GSD, 15 LN-negative GBC and 15 LN-positive GBC	KRT7, KRT19, sorcin and NPM1	KRT7 and sorcin upregulated in LNM KRT19 and NPM1—no significant results	NA

## Supplementary Materials

The following supporting information can be downloaded at https://www.mdpi.com/article/10.3390/curroncol31080333/s1. Figure S1: Molecular functions of DEPs and Reactome pathway analysis in LN vs. PT using STRING database. DEP—differentially expressed proteins; LN—lymph node; PT—primary tumor. Figure S2: Molecular functions of DEPs in LN metastatic gastrointestinal carcinoma cases using STRING database. DEP—differentially expressed proteins; LN—lymph node. Figure S3: KEGG pathway analysis of DEPs in LN metastatic gastrointestinal carcinoma cases using STRING database. DEP—differentially expressed proteins; LN—lymph node. Table S1: Differentially expressed proteins in gastrointestinal carcinomas (A) Colorectal carcinoma (B) Gastric carcinoma (C) Pancreatic Carcinoma (D) Gallbladder carcinoma. The proteomics-based studies were used for the analysis.
