# Supplementary material for: Lymph Node Metastasis in Gastrointestinal Carcinomas: A View from a Proteomics Perspective
Source: Curr Oncol. 2024 Aug 2;31(8):4455–75. doi: 10.3390/curroncol31080333 (PMC11352871; doi:10.3390/curroncol31080333)
Supplement: Supplementary file 1 [file curroncol-31-00333-s001.zip › curroncol-2992371-Supplementary Materials.pdf]

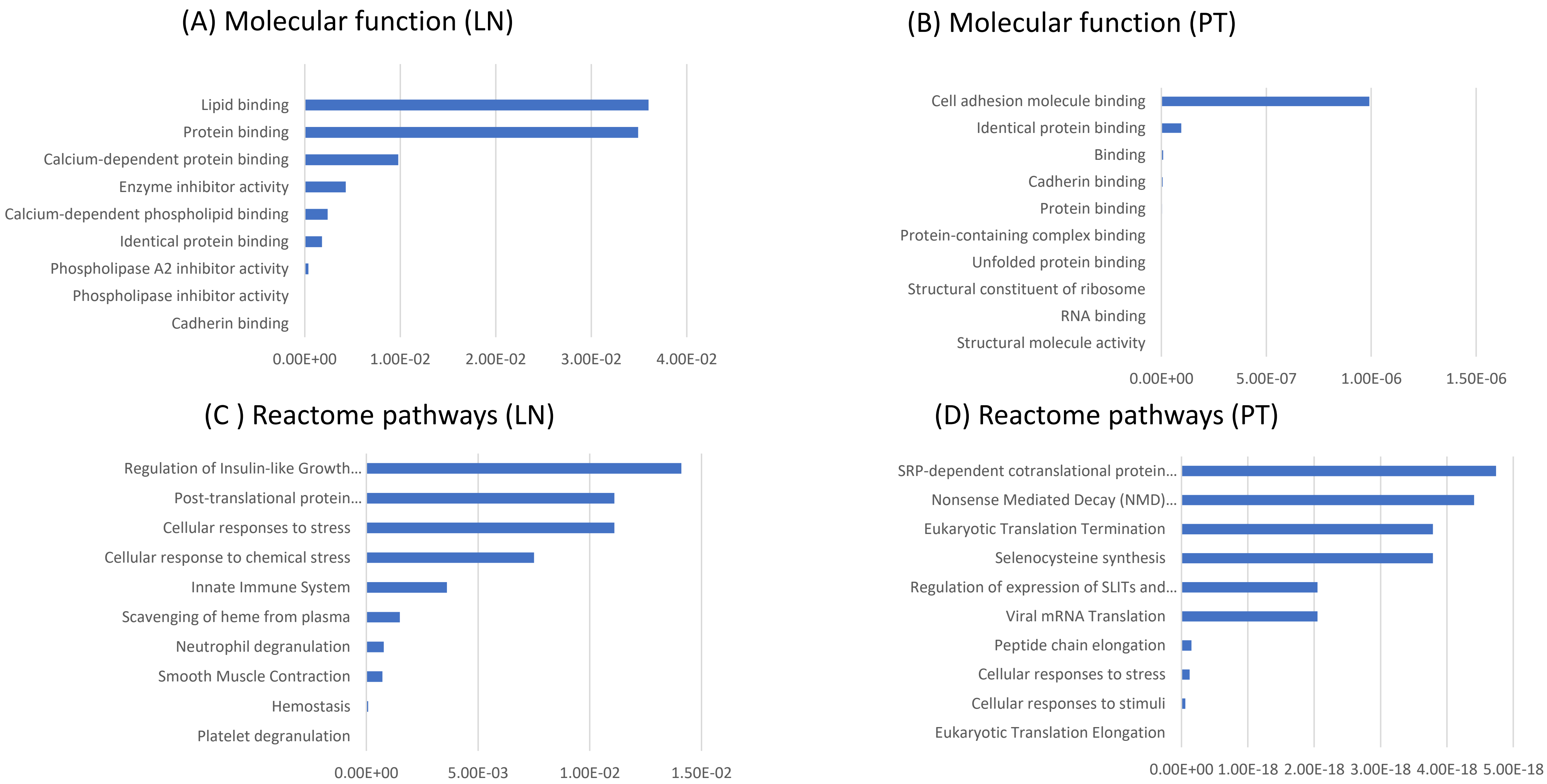

Supplementary Figure S1 : Molecular functions of DEPs and Reactome pathway analysis in LN vs PT using STRING database. DEP- Differentially expressed proteins, LN- Lymph node, PT-Primary tumor

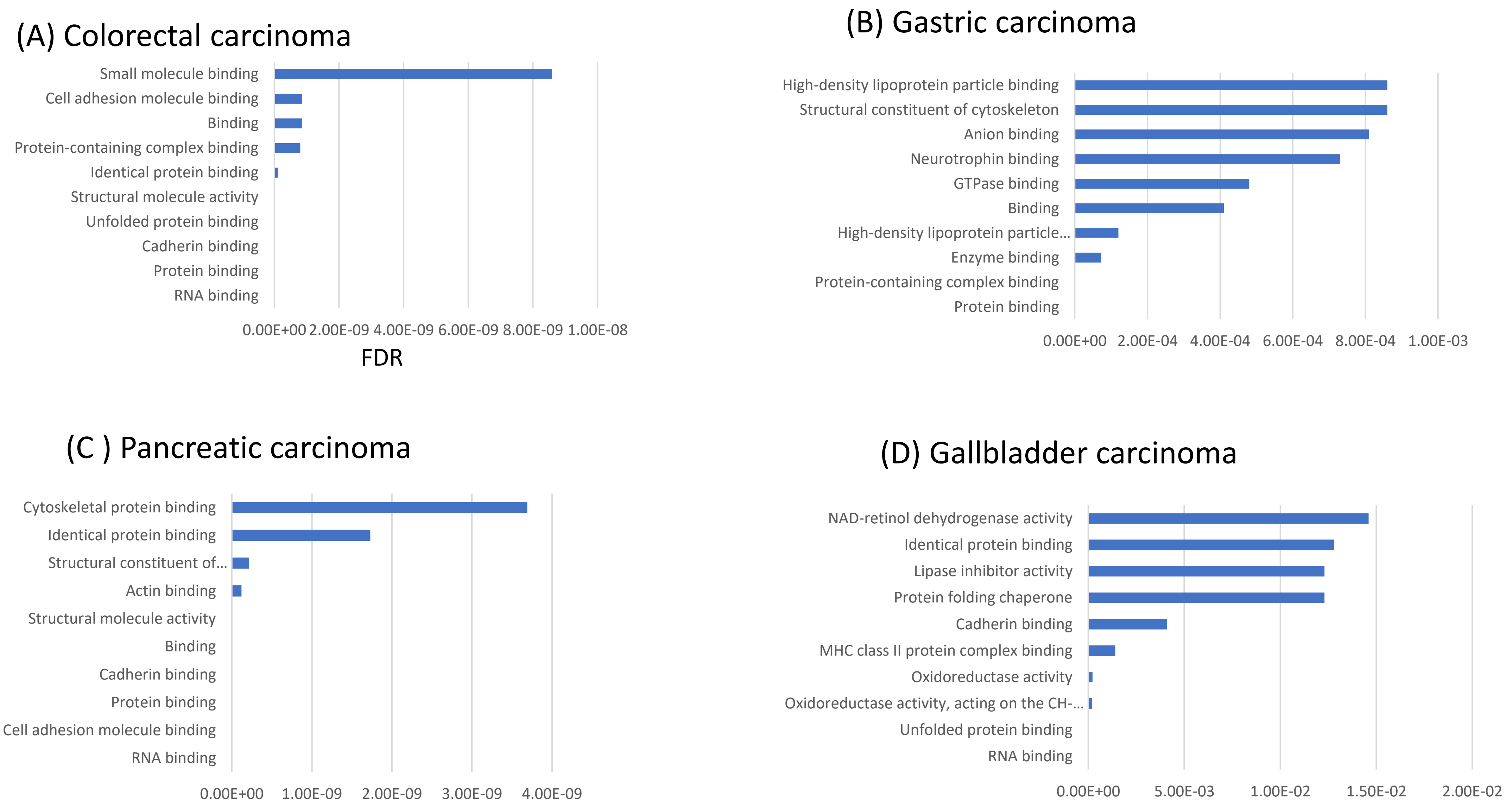

Supplementary Figure S2 : Molecular functions of DEPs in LN metastatic gastrointestinal carcinomas cases using STRING database. DEP- Differentially expressed proteins, LN- Lymph node

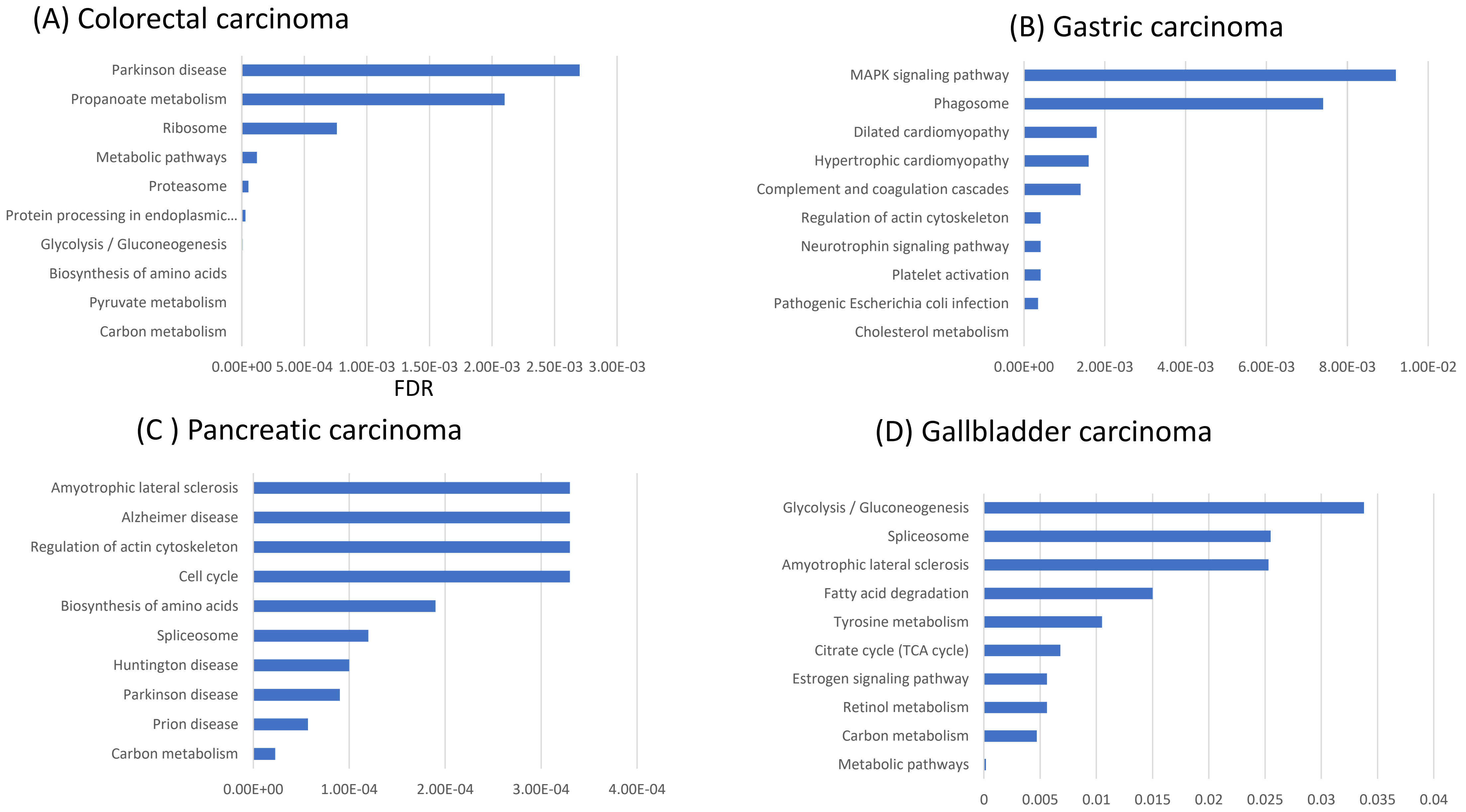

Supplementary Figure S3 : KEGG Pathway Analysis of DEPs in LN metastatic gastrointestinal carcinomas cases using STRING database. DEP- Differentially expressed proteins, LN- Lymph node
